# Impact of metformin use on neurocognitive functions in older people: a systematic review and meta-analysis

**DOI:** 10.1590/1980-5764-DN-2026-0527

**Published:** 2026-10-02

**Authors:** Madson Alan Maximiano-Barreto, Jordan Alves dos Santos Pereira, Victor José Bastos-Silva

**Affiliations:** 1 Grupo de Estudos e Pesquisas em Saúde Mental, Cognição e Envelhecimento, São Carlos SP, Brazil.; 2 Universidade de São Paulo, Departamento de Neurociências e do Comportamento, Ribeirão Preto SP, Brasil.; 3 Centro Universitário CESMAC, Grupo de Pesquisa Aplicada ao Desempenho e Saúde, Maceió AL, Brazil.

**Keywords:** Aging, Cognition, Diabetes Mellitus, Cognitive Function, Metformin, Envelhecimento, Cognição, Diabetes Mellitus, Função Cognitiva, Metformina

## Abstract

Metformin is a widely prescribed drug for managing diabetes mellitus. Recent studies have investigated its potential effects on cognitive function in adults aged 60 years and older, a population that is steadily increasing and often exhibits cognitive impairments associated with aging and other chronic conditions.

**Objective:**

This study aimed to evaluate the impact of metformin use on cognitive functions in older adults through a meta-analysis.

**Methods:**

A systematic review and meta-analysis were conducted in accordance with the PRISMA guidelines, utilizing the Embase, LILACS, PubMed, Scopus, and Web of Science databases. The review protocol was registered in PROSPERO (CRD42024624045).

**Results:**

Thirteen articles met the eligibility criteria for inclusion in this systematic review, of which five provided sufficient data for the meta-analysis. The findings indicate that metformin has a small but positive effect on overall cognitive function in older adults. Additionally, the meta-analysis demonstrated low heterogeneity (49.6%) and a low risk of bias (p>0.05).

**Conclusion:**

The results suggest that metformin may have a protective effect on the overall cognitive function of older adults, in addition to its well-established metabolic benefits.

## RESUMO

A metformina é um medicamento amplamente prescrito para o manejo da diabetes mellitus. Pesquisas recentes têm investigado seu impacto potencial na função cognitiva entre indivíduos com 60 anos ou mais, uma população que está aumentando constantemente e muitas vezes apresenta comprometimento cognitivo associado ao envelhecimento e outras condições crônicas.

**Objetivo:**

Este estudo teve como objetivo avaliar o impacto do uso da metformina nas funções cognitivas em idosos por meio de uma meta-análise.

**Métodos:**

Uma revisão sistemática e meta-análise foram conduzidas de acordo com as diretrizes PRISMA, utilizando os bancos de dados Embase, LILACS, PubMed, Scopus e Web of Science. O protocolo de revisão foi registrado no PROSPERO (CRD42024624045).

**Resultados:**

Treze artigos atenderam aos critérios de elegibilidade para inclusão nesta revisão sistemática, dos quais cinco forneceram dados suficientes para a meta-análise. Os resultados indicam que a metformina tem um efeito pequeno, mas positivo, na função cognitiva geral em idosos. Além disso, a meta-análise demonstrou baixa heterogeneidade (49,6%) e baixo risco de viés (p>0,05).

**Conclusão:**

Os resultados sugerem que a metformina pode ter um efeito protetor sobre a função cognitiva geral dos idosos, além de seus benefícios metabólicos bem estabelecidos.

## INTRODUCTION

**T**he United Nations^1^ estimates that the global population will grow from 7.7 billion in 2019 to 8.5 billion by 2030 (i.e., a 10% increase), 9.7 billion by 2050 (26%), and 10.9 billion by 2100 (42%). As the global population ages, the economic burden associated with age-related health conditions is expected to increase, underscoring the urgent need for preventive and/or therapeutic strategies^2^. Among various age-related conditions, neurodegeneration and its associated cognitive decline stand out due to their significant impact on health expectancy and quality of life^3^. Key aspects in understanding the mechanisms of aging and the pathogenesis of neurodegenerative diseases include deoxyribonucleic acid (DNA damage, mitochondrial dysfunction, cellular senescence, and inflammation^3^.

Neurodegenerative diseases encompass a heterogeneous group of neurological disorders that significantly affect millions of people worldwide (e.g., Alzheimer’s disease, Parkinson’s disease, and others)^4^. These conditions are characterized by the progressive loss of neurons in the central or peripheral nervous system^5^. The degeneration of neuronal networks, coupled with neurons’ inability to regenerate effectively due to their terminally differentiated state, disrupts essential communication pathways^5^, manifesting as cognitive impairments.

Cognition encompasses multiple domains (e.g., memory, executive function, language, social cognition, perception, and others)^6^. Impairments in these functions can result from multiple factors (e.g., low educational attainment, chronic health conditions such as diabetes)^7^. Individuals with cognitive impairment may experience several adverse consequences (e.g., reduced quality of life, impaired functional activities)^8^, often necessitating caregiving support^9^. Moreover, these impairments may lead to neurocognitive disorders (e.g., dementia due to Alzheimer’s disease)^10^.

Although advances in treating neurocognitive disorders have been reported, their benefits remain limited and often temporary^11^, as many therapies are discontinued due to toxicity or lack of efficacy^12^. However, increasing knowledge of the biological processes underlying neurodegenerative diseases, alongside advances in biomarkers and clinical characterization, has facilitated the identification of new therapeutic targets^13^ and the repurposing of existing drugs^14^, such as metformin.

Metformin (N,N-dimethylbiguanide), a biguanide class drug, comprises two linked guanidine rings and was originally derived from galegine, an isoamylene guanidine present in the French lilac (Galega officinalis)^15^. Currently, it is recommended by the American Diabetes Association as a first-line agent for type 2 diabetes management in conjunction with lifestyle modifications^16^. It is considered cost-effective and safe, given the low risk of hypoglycemia as a side effect^17^. In diabetic patients, the ideal oral dose is typically 2 g/day^15^. The off-label use of metformin in non-diabetic patients has also garnered attention for its therapeutic potential in various medical conditions, with doses of 1,600–1,700 mg/day^18^ and single doses of 500 mg used in healthy individuals to enhance physical performance^19,20,21^. After a single dose, metformin is rapidly distributed to various tissues, with partial absorption occurring in the small intestine. Intracellularly, its primary mechanism involves inhibiting complex I of the mitochondrial respiratory chain, altering cellular energy metabolism. This process increases the adenosine monophosphate/adenosine triphosphate (AMP/ATP) ratio, activating AMP-activated protein kinase (AMPK), a critical regulator of cellular functions^22^.

More recently, preclinical^23,24^ and mechanistic^25,26^ studies have expanded the understanding of metformin’s potential effects on neurodegenerative diseases. Metformin-induced AMPK activation, a key component in dementia pathogenesis^27^, has demonstrated broad neuroprotective potential. Major benefits include promoting autophagy, preserving mitochondrial quality control, reducing insulin resistance, and mitigating oxidative stress^28^. Additionally, metformin stimulates processes in the central nervous system, such as neuroprotection, neural regeneration, angiogenesis, and anti-inflammatory actions, positioning it as a promising intervention for the treatment of neurodegenerative disease^29^. Over recent decades, extensive studies have evidenced metformin’s potential benefits in conditions such as Alzheimer’s disease^30^, dementia^31^, and other neurodegenerative pathologies^32,33^.

Several clinical studies suggest that metformin may reduce the risk of Alzheimer’s disease in individuals with type 2 diabetes^34^. This protective effect appears to be mediated not only by improvements in macrovascular complications commonly associated with diabetes but also by the drug’s intrinsic neuroprotective properties. Notably, the impact of metformin on Alzheimer’s disease pathology has been reported to occur independently of glycemic control^34^, indicating a direct effect on neuronal health. Furthermore, consistent findings from preclinical studies using amyloid precursor protein/presenilin-1 (APP/PS1) mouse models have reinforced metformin’s neuroprotective potential, supporting its role in mitigating cognitive decline through mechanisms beyond glucose regulation.

A recent meta-analysis assessed the effect of metformin on neurocognitive functions^35^, concluding that metformin had no significant impact on cognition. However, this study included individuals aged 30 years and older. It is well-known that older adults (≥60 years) may experience cognitive decline with advancing age^7^, highlighting the importance of investigating this medication’s effects in older populations, an area insufficiently explored in literature. Therefore, this study aimed to conduct a systematic review and meta-analysis to evaluate metformin’s impact on cognitive function in older adults.

## METHODS

### Data sources

A systematic review was conducted following the guidelines of the Preferred Reporting Items for Systematic Reviews and Meta-Analyses (PRISMA) statement^36^. This review was registered on December 8, 2024, and approved on December 19, 2024, in the International Prospective Register of Systematic Reviews (PROSPERO; CRD42024624045). The databases Embase, LILACS, PubMed, Scopus, and Web of Science were searched using the following combination of descriptors: (“Elderly” OR “Aging” OR “Older people” OR “Older adults”) AND (“Mental processes” OR “Attention” OR “Executive function” OR “Perception” OR “Memory” OR “Social cognition” OR “Complex attention”) AND (“Metformin” OR “Dimethylbiguanidine” OR “Dimethylguanylguanidine” OR “Glucophage” OR “Metformin hydrochloride”). The database search was completed on October 31, 2024.

### Eligibility criteria

Eligibility criteria were defined *a priori*. Studies were included if they were original, quantitative, observational articles (e.g., cross-sectional, longitudinal, or case-control) that assessed the association between metformin use and neurocognitive functions in older adults (≥60 years), regardless of diabetes mellitus status, regardless of language or publication date. Exclusion criteria included validation studies, case reports, reviews, comments, notes, theses, dissertations, books, book chapters, errata, literature reviews, letters to the editor, and abstract collections.

### Data extraction

A total of 1,068 articles were identified across the databases and imported into the online reference manager Rayyan^®^ (https://www.rayyan.ai/), designed to facilitate systematic review processes^37^. Duplicate articles were identified and removed. Two authors (VJBS and JAP) independently performed an initial selection by screening titles and abstracts. Subsequently, full texts of the pre-selected articles were assessed for eligibility. Any disagreements between the two authors were initially resolved by a third author, and unresolved issues were addressed through a joint meeting to reach a final decision based on the established eligibility criteria.

To ensure comprehensive coverage, the reference lists of included articles were manually reviewed for additional relevant studies^38^, following PRISMA recommendations. All steps were conducted independently to minimize selection bias^39^. Ultimately, 13 articles were included in the systematic review, as detailed in Figure 1.

**Figure 1:**
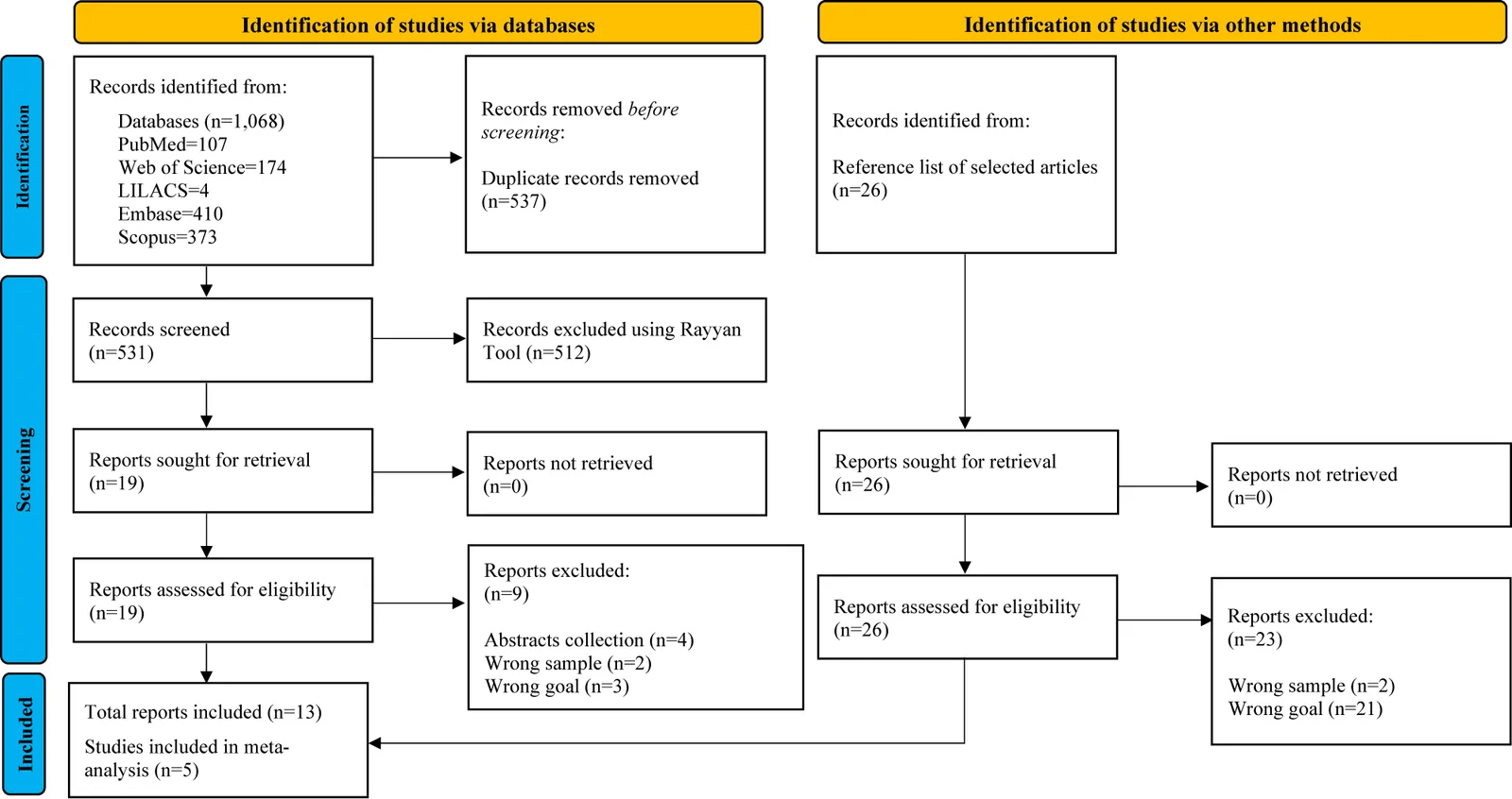
Selection of eligible articles using the Preferred Reporting Items for Systematic Reviews and Meta-Analyses flow diagram.

Source: Page et al.^36^

### Data synthesis

Key information extracted from the selected studies was organized into tables and is presented in the Results section, including:

- first author;
- year of publication;
- country where the study was conducted;
- study design;
- sex of participants;
- average age of participants;
- number of participants;
- association between metformin use and neurocognitive functions;
- dosage; and
- duration of metformin use.

Articles with missing data, except for item “association between metformin use and neurocognitive functions”, were included if they met the eligibility criteria.

### Critical appraisal

The risk of bias in the included studies was assessed using the Critical Appraisal Tools from the Joanna Briggs Institute^40^. The checklist comprises eight questions with responses rated on a Likert scale (Yes, No, Unclear, Not Applicable). Risk of bias was classified based on the percentage of “Yes” responses: ≥70% (low risk), 50–69% (moderate risk), and <50% (high risk). This classification aligns with previous systematic reviews^41,42^. The evaluation was conducted independently, and disagreements were resolved during a consensus meeting involving three authors.

### Statistical analysis

A meta-analysis was conducted to evaluate the standardized mean difference (SMD) in global cognition scores between older adults using and not using metformin. Because global cognition was assessed using different cognitive instruments across studies, the SMD was selected as the summary measure of effect, allowing comparisons across different scales. Of the studies included in the systematic review, only those reporting sufficient quantitative data on global cognitive performance for both metformin users and comparison groups were eligible for meta-analysis. The included studies comprised populations with diabetes mellitus, as well as mixed samples including participants without diabetes mellitus. Effect sizes were categorized as small (0.2), medium (0.5), and large (0.8) based on Cohen’s guidelines^43^. The DerSimonian-Laird estimator was used to assess heterogeneity^44^. Funnel plots were generated to evaluate potential bias, with asymmetry assessed using the Egger and Begg tests^45,46^. All statistical analyses were performed using Jamovi software (version 2.3).

## RESULTS

### Study selection

A total of 1,068 articles were identified across the databases. Following the screening of titles and abstracts, 531 articles were evaluated, with 19 selected for full-text review. Of these, nine articles were excluded for not meeting the eligibility criteria. Subsequently, ten articles were included in the review. Additionally, a reference list search of these articles identified three more studies that met the inclusion criteria. Thus, 13 articles were included in this systematic review. Among these, five provided the required data (i.e., means and standard deviations of cognitive measures for older adults using and not using metformin) for meta-analysis. The main results are summarized in Tables 1 and 2 ^30,31,32,33,47,48,49,50,51,52,53,54,55^.

**Table 1:** Characteristics of the 13 selected studies.

Author	Year	Country	Design	Sample	Sex (%)	Age (year) ( $\bar{\text{X}}$ ±SD)	Diabetics/nondiabetics (%)
Sood et al.^32^	2024	United States	Longitudinal	3,029	F (72.0)	78.7±7.4	Diabetics (11.0) Nondiabetics (89.0)
Borzì et al.^47^	2019	Italy	Longitudinal	60	F (55.0)	76.5±7.8	Diabetics (100.0)
Isik et al.^48^	2017	Turkey	Longitudinal	205	F (60.0)	75.3±7.5	Diabetics (100.0)
Koo et al.^49^	2019	South Korea	Longitudinal	732	F (67.6)	76.6±6.7	Diabetics (21.4) Nondiabetics (78.6)
Mone et al.^50^	2021	Italy	Case-control	209	-	75.5±5.9	Diabetics (75.6) Nondiabetics (24.4)
Pomilio et al.^30^	2022	United States Europe Japan Korea China India Argentina	Case-control	810	F (39.9)	73.4±6.2	Diabetics (8.9) Nondiabetics (91.1)
Samaras et al.^31^	2020	Australia	Longitudinal	1,037	F (54.7)	78.8±7.7	Diabetics (12.0) Nondiabetics (88.0)
Santulli et al.^51^	2024	Italy	Case-control	208	F (55.3)	78.5±8.5	Diabetics (49.5) Nondiabetics (50.5)
Soldevila-Domenech et al.^52^	2021	Italy	Longitudinal	487	F (50.5)	65.2±4.7	Diabetics (30.4) Nondiabetics (69.6)
Teng et al.^53^	2021	China	Case-control	234	F (46.2)	67.8±8.9	Diabetics (100.0)
Weinstein et al.^54^	2019	United States Iceland the Netherlands	Cross-sectional	3,590	F (54.4)	67.8±7.9	Diabetics (100.0)
Wu et al.^33^	2020	United States	Longitudinal	2,670	F (52.2)	73.9±8.3	Diabetics (100.0)
Xue et al.^55^	2023	United States	Case-control	1,306	-	75.47	Diabetics (100.0)

Abbreviations: F, Female; $\bar{\text{X}}$ : mean; SD, Standard Deviation; -, Not described.

**Table 2:** Effect of metformin on cognition.

Author	Sample in use of metformin (%)	Sample in use of another drug/placebo (dose)	Treatment dose (metformin)	Duration (metformin)	Measure neurocognitive functions	Outcome
Sood et al.^32^	(8.7)	Insulin	-	-	MMSE: Global cognition	Slower decrease of global cognition; episodic and semantic memory
Borzì et al.^47^	(50.0)	Metformin (2,000 mg/day) + Vildagliptin (100 mg/day)	2,000 mg/day	1 month	MMSE: Global cognition	Cognitive decline in the metformin-only group; preservation of cognition in the metformin + vildagliptin group
Isik et al.^48^	(33.6)	Sitagliptin (100 mg/day) Insulin	-	6 months	MMSE: Global cognition	Metformin had worse performance in cognition when compared to other drugs (i.e., sitagliptin or insulin)
Koo et al.^49^	(59.2)	-	-	35 months	MMSE: Global cognition IVR: Verbal learning VFT: Verbal fluency BNT: Naming	Lower scores in global cognition and immediate and delayed verbal learning
Mone et al.^50^	(25.3)	Insulin	-	-	MoCA: Global cognition	Hyperglycemic patients treated with metformin showed significantly higher MoCA scores than those treated with insulin
Pomilio et al.^30^	(8.9)	-	-	104 months	MMSE: Global cognition RAVLT: Episodic memory ADAS-Cog: Global cognition TMTA: Attention: CVLT: Verbal memory IVR: Immediate memory CP: Constructive visuospatial skills DFVR: Long-term verbal memory DVRCR: Long-term memory and visual and spatial memory	Older adults using metformin showed better cognitive performance
Samaras et al.^31^	(3.3)	-	-	12 months to 10 years	RAVLT: Episodic memory LMS: Episodic memory BVRT: Visual memory WAIS-III: Visuospatial Skill TMT: Attention and cognitive flexibility BNT: Naming CFT: Verbal fluency WAIS-BD: Visuospatial skill LFT: Verbal fluency	Slower decrease in overall cognition and executive function compared to those who did not use metformin
Santulli et al.^51^	(10.6)	-	500 mg/day	6 months	MoCA: Global Cognition	-
Soldevila-Domenech et al.^52^	(17.9)	-	-	1 to 3 years	RAVLT: Episodic memory RCFT: Attention visuoconstructive abilities, memory, short- and long-term visual perception SDMT: Processing speed SCWIT: Inhibition and attention IGT: Decision making CPT: Inattention, impulsiveness, sustained attention and vigilance MMSE: Global cognition	Older people on metformin had better performance in global cognition, episodic memory, and executive functions. Evaluation after three years showed opposite result.
Teng et al.^53^	(100.0)	-	-	>6 years <6 years	MMSE: Global cognition	Older people on prolonged metformin use had reduced cognitive function
Weinstein et al.^54^	-	Sulfonylurea Insulin	-	-	TMTA: Attention TMT: Attention and cognitive flexibility LMS: episodic memory	No significant association was observed with global cognition, executive function, or memory performance.
Wu et al.^33^	(65.3)	-	-	-	MMSE: Global cognition WMS-III: Memory WMS-R: Logic memory	Older people using metformin showed better memory performance
Xue et al.^55^	(60.1)	-	-	-	MMSE: Global cognition	Metformin use does not correlate with the risk of developing severe dementia in older adults

Abbreviations: ADAS-Cog, Alzheimer’s Disease Assessment Scale-Cognitive Subscale; BNT, Boston Naming Test; BVRT, Benton Visual Retention Test; CFT, Category Fluency Test; CP, Constructional Praxis; CPT, Conners’ Continuous Performance Test; CVLT, California Verbal Learning Test; DVRCR, Delayed Verbal Recognition and Constructive Recall; IGT, Iowa Gambling Task; IVR, Immediate Verbal Recall; LFT, Letter Fluency Test; LMS, Logical Memory Story; MMSE, Mini-Mental State Examination; MoCA, Montreal Cognitive Assessment; RAVLT, Rey Auditory Verbal Learning Test; RCFT, Rey-Osterrieth Complex Figure Test; DFVR, Delayed Free Verbal Recall; SCWIT, Stroop Color-Word Interference Test; SDMT, Symbol Digit Modalities Test; TMT, Trail Making Test; TMTA, Trail Making Test A; VFT, Verbal Fluency Test; WAIS-BD, Block Design from the Wechsler Adult Intelligence Scale; WAIS-III, Wechsler Adult Intelligence Scale-III Digit-Symbol-Coding; WMS-III, Wechsler Memory Scale; WMS-R, Revised Wechsler Memory Scale; -, Not described.

### Quality of studies

The risk of bias assessment, detailed in Table 3 ^30,31,32,33,47,48,49,50,51,52,53,54,55^, revealed a low risk across all studies, with percentages ranging from 75 to 100%. Six studies achieved a score of 100%^30,31,33,51,53,54^, indicating high methodological quality. Two studies scored 87.5%^32,52^, while the remaining studies scored 75%^47,48,49,50,55^. Lower scores were attributed to a lack of identification and control of confounding variables (e.g., educational attainment). The risk of bias checklist for cross-sectional studies was also applied to the baseline assessment of other study designs (e.g., longitudinal).

**Table 3:** Assessment of the risk of bias in the articles included in this systematic review.

Author	Critical appraisal tools items								Total score	Total (%)
	1	2	3	4	5	6	7	8		
Sood et al.^32^	?	+	+	+	+	+	+	+	7/8	87.5
Borzì et al.^47^	+	+	+	+	-	-	+	+	6/8	75.0
Isik et al.^48^	+	+	+	+	-	-	+	+	6/8	75.0
Koo et al.^49^	+	+	+	+	-	-	+	+	6/8	75.0
Mone et al.^50^	+	+	+	+	-	-	+	+	6/8	75.0
Pomilio et al.^30^	+	+	+	+	+	+	+	+	8/8	100.0
Samaras et al.^31^	+	+	+	+	+	+	+	+	8/8	100.0
Santulli et al.^51^	+	+	+	+	+	+	+	+	8/8	100.0
Soldevila-Domenech et al.^52^	?	+	+	+	+	+	+	+	7/8	87.5
Teng et al.^53^	+	+	+	+	+	+	+	+	8/8	100.0
Weinstein et al.^54^	+	+	+	+	+	+	+	+	8/8	100.0
Wu et al.^33^	+	+	+	+	+	+	+	+	8/8	100.0
Xue et al.^55^	+	+	+	+	-	-	+	+	6/8	75.0

Notes: +:Yes. −: No. ?: Unclear. Items: 1. Were the criteria for inclusion in the sample clearly defined? 2. Were the study subjects and settings described in detail? 3. Was the exposure measured in a valid and reliable way? 4. Were objective, standard criteria used for measurement of the condition? 5. Were confounding factors identified? 6. Were strategies to deal with confounding factors stated? 7. Were the outcomes measured in a valid and reliable way? 8. Was appropriate statistical analysis used?

### Study characteristics

The selected studies were published between 2016^48^ and 2024^32,51^, encompassing research conducted across four continents: the Americas, Asia, Europe, and Oceania. Studies in the Americas were primarily conducted in the United States^30,32,33,54,55^, with some including participants from other regions (e.g., Japan, China, India, Argentina, Iceland). Asian studies were conducted in South Korea^49^, China^53^, and Turkey^48^. European research was predominantly based in Italy^49,50,51,55^, while Australia represented Oceania^31^.

Most studies employed longitudinal designs^32,48,49,55^, followed by case-control^30,50,51,53,55^ and one cross-sectional design^54^. The combined sample included 10,500 older adults, with sample sizes ranging from 60^55^ to 3,590 participants^54^.

The majority of studies reported a female prevalence exceeding 50%^31,32,33,48,51,52,53,54,55^, with frequencies ranging from 39.9^30^ to 74.3%^54^. Two studies did not report this information^50,55^. Most participants had a mean age above 70 years, ranging from 65.2 years (standard deviation ±4.7)^52^ to 78.8 (±7.8)^31^.

### Metformin dosage and duration of use

Information on metformin dosage was absent in most studies^30,31,32,33,48,49,50,51,52,53,54,55^. For studies that provided dosage data, values ranged from 500 mg/day^51^ to 2,000 mg/day^47^. Usage durations varied from six months^48,51^ to ten years^31^.

### Cognitive function evaluation

Various instruments were utilized to assess neurocognitive domains (e.g., memory, executive function), with global measures being most prevalent. The Mini-Mental State Examination (MMSE) was employed in most studies^30,32,48,49,52,55^. The Montreal Cognitive Assessment (MoCA) was the second most commonly used tool^49,50^.

### Effect of metformin on cognitive function

The qualitative synthesis yielded both positive and negative findings regarding the effects of metformin, warranting a meta-analysis. Five studies were included in the meta-analysis^30,47,48,50,55^, which indicated a small but positive effect of metformin on overall cognitive function (SMD=0.23, p=0.019; 95% confidence interval [CI] 0.04–0.42; I^2^=49.26%; Q=.096), suggesting low heterogeneity. Egger’s and Begg’s tests confirmed the absence of publication bias (p=1.000 and p=0.963, respectively), as illustrated in Figures 2 and 3.

**Figure 2:**
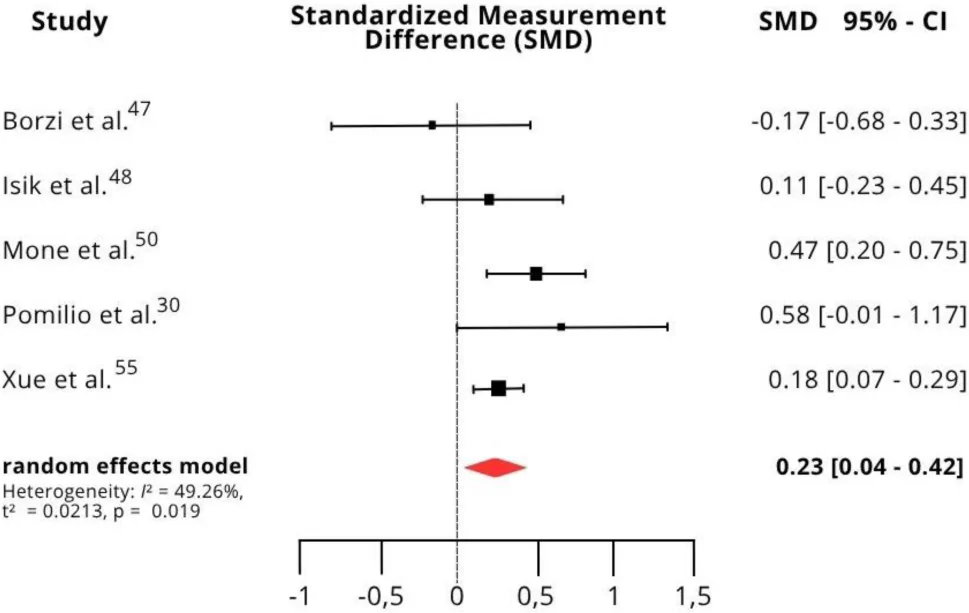
Forest plot of the effect of metformin on global cognition in older people.

**Figure 3:**
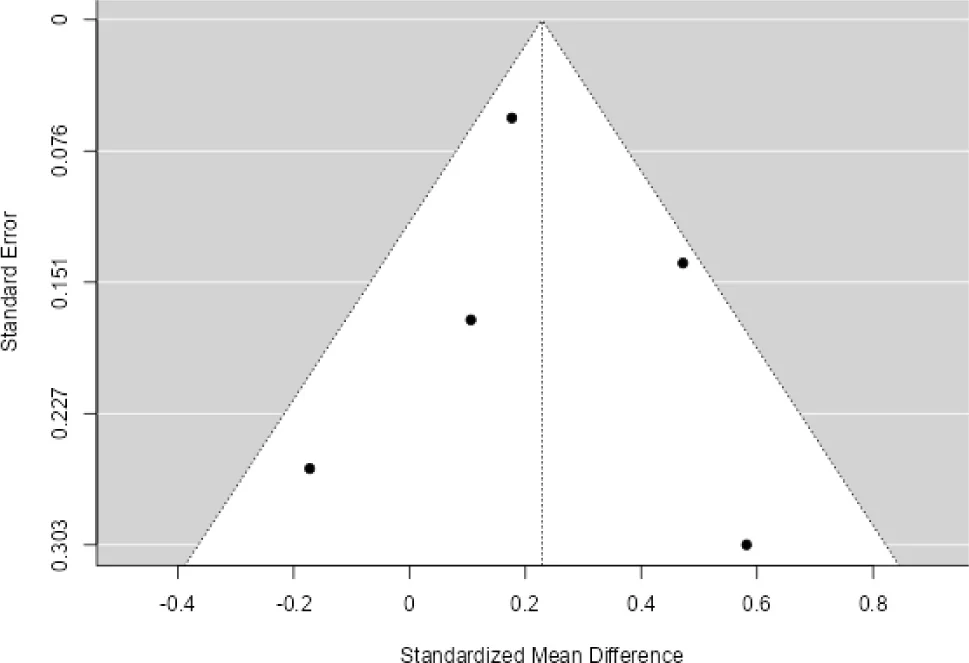
Funnel chart to identify bias.

Figure 4 summarizes the main findings of this review and illustrates the proposed mechanisms through which metformin may influence cognitive function in older adults.

**Figure 4:**
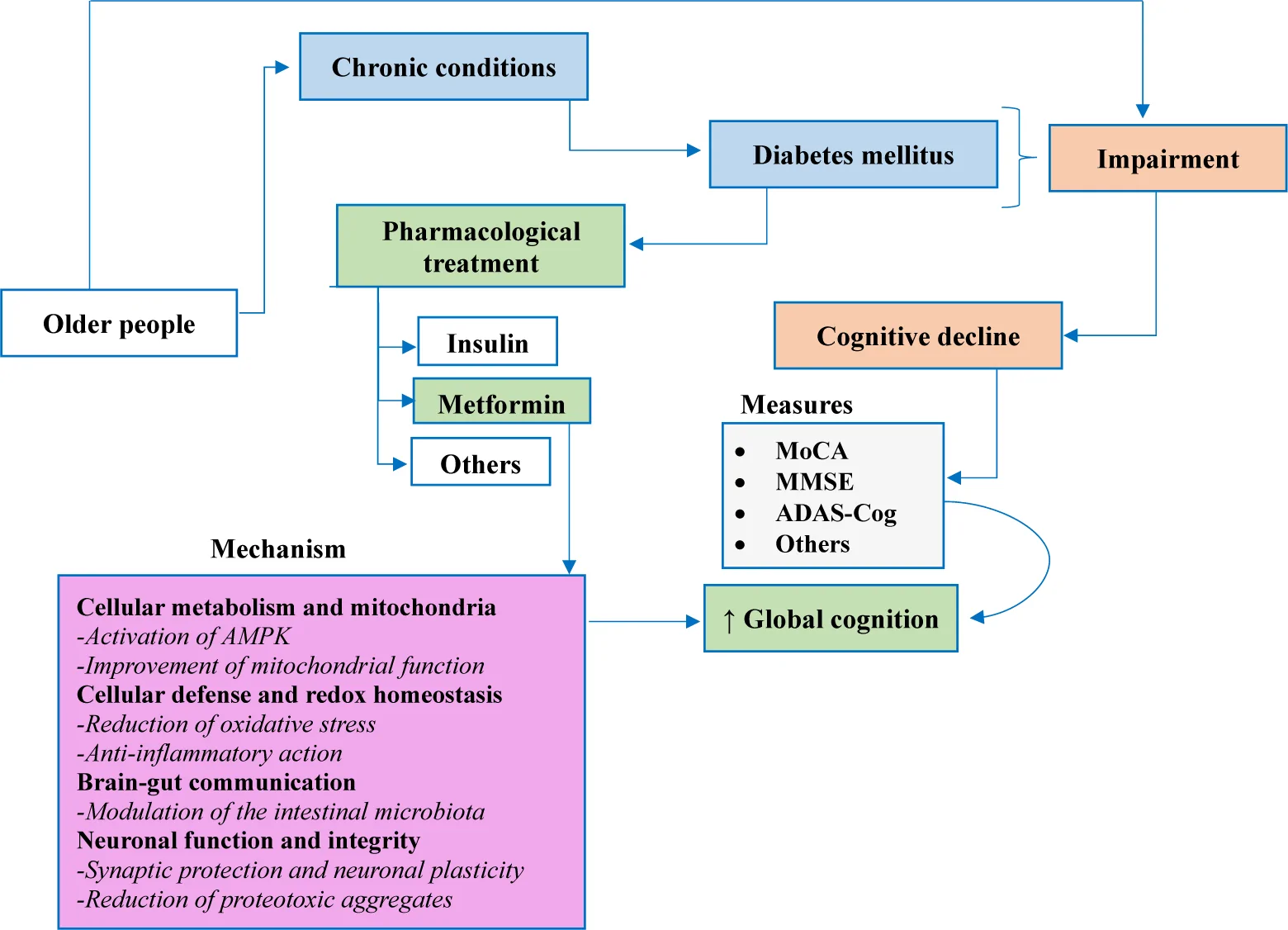
Summary of the effect of metformin on global cognition of older people with diabetes and its main mechanism.

Note: ↑: Higher.

Abbreviations: ADAS-Cog, Disease Assessment Scale-Cognitive; MMSE, Mini-Mental State Examination; MoCA, Montreal Cognitive Assessment.

## DISCUSSION

This systematic review with meta-analysis investigated the impact of metformin use on neurocognitive functions in older adults, as reported in the literature. The main findings indicate that metformin may exert a small but positive effect on overall neurocognitive function in individuals aged ≥60 years.

The findings differ from those reported in the previous systematic review by Malazy et al.^35^, which also examined the impact of metformin on cognitive function. This discrepancy may be partly explained by differences in the age ranges of the populations included in the respective reviews. While the present review focused exclusively on individuals aged ≥60 years, a group particularly vulnerable to age-related cognitive decline^56^, the review by Malazy et al.^35^ included a broader age range, starting from 30 years old. This age distinction could have influenced the findings, given the more pronounced impact of aging on neurocognitive functions in populations aged ≥60 years

Cognitive decline with aging is shaped by a complex interplay of biological, lifestyle, and environmental factors^57^. Physiological brain aging manifests as reduced cognitive, social, and motor abilities, including slower information processing, impaired executive functions, reduced planning capability, and diminished working memory^57^. Furthermore, conditions such as Parkinson’s disease and cerebrovascular diseases contribute to cognitive deterioration through distinct pathophysiological mechanisms^58^. Inadequate lifestyle habits in older adults, such as physical inactivity, poor diet, smoking, and excessive alcohol consumption, also significantly impair cognitive performance^58^.

In this context, the findings suggest that metformin may offer particularly relevant benefits to individuals aged ≥60 years, possibly due to its effects on metabolic pathways associated with aging and neuroprotection^59^. Key mechanisms include modulation of insulin resistance and reduction of inflammatory processes linked to neurodegeneration^60,61^. Inflammation plays a critical role in diabetes-related cognitive dysfunction, as chronic hyperglycemia activates the NOD-like receptor protein 3 (NLRP3) inflammasome and triggers inflammatory cascades that impair cognition. Microglia, key regulators of neuroinflammation, contribute to neuronal apoptosis and cognitive decline when overactivated, often due to metabolic dysfunction. Hyperglycemia exacerbates microglial reactivity via sterol regulatory element-binding protein cleavage activating protein (SCAP)-mediated upregulation of NLRP3 and inflammatory cytokines. These cells express phosphoinositide 3-kinase (PI3K), which modulates synaptic plasticity and inflammation through the PI3K/ protein kinase B (AKT) pathway, influencing oxidative stress, neuroinflammation, and brain-derived neurotrophic factor (BDNF) signaling. Microglia polarize into proinflammatory M1 (tumor necrosis factor-α and interleukin [IL]-6) or anti-inflammatory M2 (secreting IL-4, IL-10) phenotypes, with Tregs critically determining polarization. Impaired Tregs drive M1 dominance, exacerbating neuroinflammation and cognitive dysfunction^62^.

To further explore potential mechanisms underlying these observations, recent evidence has shown that metformin can mitigate oxidative stress induced by hyperglycemia in vascular cells. In human coronary artery smooth muscle cells and endothelial cells — both implicated in the pathophysiology of ischemia with non-obstructive coronary artery — metformin significantly attenuated the increased mitochondrial production of reactive oxygen species (ROS) triggered by high glucose concentrations^62^. These findings suggest that metformin’s ability to reduce mitochondrial oxidative stress may be a key factor in its neuroprotective and anti-inflammatory effects, particularly in the context of diabetes-related metabolic dysfunction.

This reduction in oxidative stress may be closely linked to metformin’s direct action on mitochondrial respiration. Oxidative stress is a key contributor to diabetes-related cognitive dysfunction, occurring when hyperglycemia disrupts the balance between oxidants and antioxidants^62^. Excess glucose metabolism elevates ROS, depletes antioxidant defenses (e.g., superoxide dismutase, reduced glutathione), and damages brain tissue through lipid peroxidation and nicotinamide adenine dinucleotide phosphate (NADPH) oxidase activation. This stress impairs the nuclear factor erythroid 2-related factor 2/heme oxygenase-1/NAD(P)H quinone oxidoreductase 1 (Nrf2/HO-1/NQO1) pathway, further reducing antioxidant capacity, while also increasing acetylcholinesterase activity and disrupting neuronal/synaptic plasticity^63^. Mitochondrial ROS overproduction and protein glycosylation exacerbate these effects, collectively driving cognitive decline in diabetes. In this context, metformin’s inhibition of complex I in the mitochondrial respiratory chain reduces ATP synthesis and increases AMP and adenosine diphosphate (ADP) levels, leading to activation of AMPK — a central regulator of energy homeostasis. AMPK activation is strongly associated with reduced oxidative stress and inflammation, reinforcing metformin’s role as a neuroprotective agent in metabolic and neurodegenerative disorders^64,65^.

In line with these mechanisms, excessive energy intake — especially through high-fat and high-sugar diets — has been shown to suppress the expression of key neuroprotective factors such as BDNF, peroxisome proliferator-activated receptor gamma coactivator 1-alpha (PGC-1α), and sirtuin 1 (SIRT1), thereby impairing cognitive function and promoting cellular damage^66^. In animal models, such diets accelerate brain aging by exacerbating oxidative stress, neuroinflammation, and impairing autophagic processes. Among the sirtuins, SIRT1 functions as a critical nicotinamide adenine dinucleotide (NAD^+^)-dependent metabolic sensor involved in DNA repair, inflammation modulation, and cellular stress responses. Its activation, typically triggered by caloric restriction or exercise, leads to enhanced mitochondrial biogenesis and neuronal survival via regulation of transcription factors such as forkhead box O (FOXO) and coactivators like PGC-1α, while concurrently suppressing nuclear factor kappa B (NF-κB)-driven inflammatory signaling^67^. Importantly, metformin has been shown to directly enhance SIRT1 activity, promoting deacetylation of peroxisome proliferator-activated receptor gamma coactivator 1-alpha (PGC-1α) and thereby improving mitochondrial function and insulin sensitivity. These actions are further potentiated by AMPK activation, reinforcing a metabolic profile associated with cognitive protection. Supporting this translational relevance, metformin use has been linked to favorable cognitive trajectories in large cohort studies involving commonly prescribed drugs such as atorvastatin, simvastatin, warfarin, cholinesterase inhibitors, and zopiclone^68^. Moreover, the ongoing MET-FINGER clinical trial is actively investigating metformin’s therapeutic potential in preventing dementia among at-risk populations. Corroborating these findings, experimental models of Parkinson’s disease have demonstrated that metformin-induced activation of the AMPK–FOXO3 axis can lead to improvements in motor function and significant reductions in oxidative stress markers^69^, highlighting the broader neuroprotective promise of this agent across neurodegenerative conditions.

Building on this complex interplay between metabolic dysfunction and neurodegeneration, recent evidence has highlighted the gut microbiota as a key player in the pathogenesis of diabetes-related cognitive impairment. Through the microbiota-gut-brain axis, intestinal microbes influence host physiology by producing metabolites and modulating immune responses that impact brain function^62^. This bidirectional communication occurs via multiple mechanisms: circulating cytokines such as IL-6, microbial metabolites like acetate that modulate hippocampal synaptic function through vagus nerve stimulation, and specific taxa such as *Akkermansia muciniphila*, which has been shown to reduce neuroinflammation^62^. Dysregulation of these pathways — particularly decreased acetate levels and elevated IL-6 — has been implicated in synaptic dysfunction and cognitive decline in diabetes. In this context, metformin’s emerging role in modulating gut microbiota composition offers an additional neuroprotective mechanism. Human studies have suggested that metformin may enhance memory, semantic memory, and executive function, potentially through shifts in microbiota profiles and microbial-host co-metabolite production^70^. Although the precise mechanisms remain to be fully elucidated, these findings underscore the potential of metformin not only as a metabolic regulator but also as a modulator of neuroimmune and neurochemical signaling via the gut-brain axis.

Extending these multifaceted neuroprotective actions, metformin has also demonstrated the ability to restore mitochondrial homeostasis by reducing mitochondrial DNA release — a key trigger of the cyclic GMP-AMP synthase-stimulator of interferon genes (cGAS-STING) signaling pathway, which promotes astrocyte senescence and neurodegeneration. By inhibiting this pathway, metformin delays cellular aging in neural tissue, with mitofusin 2 (Mfn2) playing a pivotal role in preserving mitochondrial integrity^71^. These effects complement earlier evidence of its antioxidative and anti-inflammatory properties. Furthermore, metformin has been shown to activate chaperone-mediated autophagy (CMA) through the transforming growth factor-β-activated kinase 1– IκB kinase α/β (TAK1–IKKα/β) signaling axis, facilitating the degradation of APP. This mechanism contributes to the reduction of β-amyloid plaque formation and amelioration of cognitive deficits in Alzheimer’s disease models^72^, reinforcing the therapeutic potential of metformin across diverse neurodegenerative pathways.

Altogether, these findings support a growing body of evidence suggesting a protective role of metformin in preserving cognitive function. Observational data indicate that individuals using metformin may experience a significantly lower risk of developing dementia and mild cognitive impairment, with best estimates pointing to a 23% reduction in incident dementia and a 17% reduction in mild cognitive impairment compared to those on other glucose-lowering therapies^73^. Such benefits appear most prominent in key cognitive domains among older adults without obesity, further emphasizing the interaction between glycemic regulation and brain function^74^. Favorable cognitive outcomes have also been observed with the use of extended-release metformin in frail women with diabetes and hypertension, highlighting its potential applicability in vulnerable populations^75^. Nonetheless, while the accumulated evidence is promising, the need for larger, well-controlled prospective studies remains critical to confirm the cognitive safety and therapeutic relevance of metformin in diverse clinical settings^76^.

Building upon the growing recognition of metformin’s neuroprotective properties across metabolic, inflammatory, mitochondrial, and gut-mediated pathways, recent research has begun to expand its potential applications beyond glycemic control in diabetes. Unlike earlier findings that primarily linked metformin’s cognitive benefits to its anti-hyperglycemic effects and restricted its use to diabetic populations^77,78^, emerging evidence points to its relevance in psychiatric contexts as well. For instance, in patients with schizophrenia undergoing antipsychotic treatment, metformin has demonstrated improvements in cognitive performance^79^. Additionally, it has shown promise as an adjunctive therapy for enhancing memory and overall cognitive function^80^. These results broaden the therapeutic landscape of metformin, suggesting that its cognitive benefits may stem from pleiotropic neuroprotective mechanisms with potential utility across a variety of clinical conditions.

Thus, metformin emerges as a promising candidate for the prevention and management of neurodegenerative disorders. Qualitative analysis (Table 2) indicates that the drug appears to reduce cognitive impairment or slow cognitive decline, potentially explaining the small effect size of 0.23 observed. Although this effect is considered small, comparisons with other therapies reinforce metformin’s relevance as a more appropriate prescription option^81^. This suitability is particularly significant for older populations (≥60 years), who are especially vulnerable to cognitive decline^58^, and for whom diabetes is an additional risk factor^82^. In this context, metformin not only offers metabolic benefits but also potential neuroprotective advantages, enhancing its therapeutic implications for this age group.

This systematic review and meta-analysis identified an effect of metformin on global cognition of older adults; however, several limitations warrant discussion. The relatively small number of studies identified in the databases represents an important limitation. Furthermore, the literature search was conducted exclusively using English-language descriptors, potentially excluding studies published in other languages (e.g., Portuguese or Spanish), which may have limited the inclusion of research conducted in developing countries. Another significant limitation is the scarcity of studies investigating the effects of metformin on specific cognitive domains, such as memory, executive function, and social cognition. An additional limitation of the meta-analysis is the incomplete and heterogeneous reporting of metformin dosage and treatment duration across studies. Because these variables were inconsistently reported, subgroup analyses or meta-regression according to dosage or duration of use were not feasible. Moreover, insufficient information regarding comparator treatment dosages further limited the assessment of potential dose-response relationships. Therefore, the pooled estimates should be interpreted with caution. Despite these limitations, the findings emphasize the importance of further research, particularly longitudinal studies and intervention trials, to better understand the impact of metformin on global cognition and specific neurocognitive functions in older adults.

In conclusion, this systematic review and meta-analysis evaluated the effects of metformin on cognitive function in older adults. The primary finding suggests that metformin may exert a protective effect on global cognition in this population. These findings highlight the potential of metformin as a neuroprotective intervention, particularly due to its multifaceted mechanisms of action, including activation of AMPK, normalization of mitochondrial function, and induction of autophagy. These processes may counteract key contributors to cognitive decline, such as oxidative stress, inflammation, and the accumulation of neurotoxic proteins. Moreover, focusing on individuals aged ≥60 years provides valuable insights into the interaction between metabolic interventions, aging, and cognitive decline. These findings also support the development of future clinical trials involving cognitively healthy older adults.

## Data Availability

The datasets generated and/or analyzed during the current study are available from the corresponding author upon reasonable request.
